# The association of pathogenic factors of metabolic syndrome on serum prostate-specific antigen levels: a pilot study

**DOI:** 10.1186/s12894-019-0549-2

**Published:** 2019-11-21

**Authors:** Bo-Wen Xia, Si-Cong Zhao, Zong-Ping Chen, Chao Chen, Tian-Shu Liu, Fan Yang, Yong Yan

**Affiliations:** Department of Urology, Beijing Shijitan Hospital, Capital Medical University, 10th Tieyi Road, Haidian District, Beijing, 100038 China

**Keywords:** Metabolic syndrome, Prostate-specific antigen, Insulin resistance, Sex hormone binding globulin, Screening

## Abstract

**Background:**

Metabolic syndrome (MetS) and serum prostate-specific antigen (PSA) levels are correlated. To investigate the underlying effect of MetS on PSA levels, the relationship between the major pathogenic factors of MetS and serum PSA levels was studied.

**Methods:**

A total of 506 ostensibly healthy men who underwent routine health check-ups were recruited to this study. We evaluated the effect of the major pathogenic factors of MetS, which included insulin resistance, a subclinical inflammatory state and sexual hormone changes, on serum PSA levels by using linear regression analysis and multivariate analysis after adjusting for age, BMI and prostate volume.

**Results:**

When simultaneously adjusting for age, BMI, prostate volume and high-density lipoprotein cholesterol, serum insulin levels and SHBG levels were inversely correlated with serum PSA levels (*P* = 0.049 and *P* = 0.004, respectively), and testosterone levels were positively correlated with serum PSA levels (*P* = 0.039). In multivariate regression models, serum insulin levels and serum SHBG levels were significantly associated with serum PSA levels (both *P* < 0.001).

**Conclusions:**

Among the major pathogenic factors of metabolic syndrome, insulin resistance and sexual hormone changes may be the most significant contributors to the decline in serum PSA levels.

## Background

Prostate cancer (PCa) is the second most common cancer in the world, with nearly 1.3 million new cases and neerly 0.4 million PCa-associated deaths worldwide in 2018, and becoming the 5th leading cause of cancer-associated death in men [1]. Serum prostate-specific antigen (PSA) testing has been take for worldwide as a screening and diagnostic tool for PCa management [2]; however, the sensitivity and specificity of serum PSA levels are limited, as many factors, for instance age, obesity, prostate volume (PV), and benign prostate diseases, may significantly affect PSA levels [3].

Metabolic syndrome (MetS) is considered as a complicated clinical disease characterized by a combination of multiple metabolic disorders, which include obesity, hypertension, dyslipidemia, hyperglycemia and insulin resistance [4]. MetS is prevalent worldwide and has become a major social and public health issue in the last 2 decades. Previous studies demonstrated that MetS may be involved in the onset and progression of certain types of cancer including carcinoma of the liver, colon, breast, bladder, prostate, etc. [5]. Additionally, studies that aimed to improve PCa management have also demonstrated the specific relationship between MetS and PSA levels [6–8]. Our previous study made investigate the association between MetS and serum PSA levels. We first found that the diagnosis of MetS decreased serum PSA levels by 11.3% compared to the absence of MetS after adjustment with a larger PV in MetS patients, at the same time, there is a linear relationship between the decrease of PSA and the number of components of metabolic syndrome [9].

However, the detailed effect of MetS on PSA levels is still unclear. In the recent period of time, increasing evidence has suggested insulin resistance (IR), a subclinical inflammatory state and sex hormone changes may be the major pathogenic factors involved in MetS [4]. Historically, IR was considered the key feature of MetS, and the effect of IR significantly increased the prevalence of MetS [10].

Second, previous studies clearly demonstrated that there was a causal relationship between changes in serum sex hormone levels and MetS, as MetS was accompanied by sex hormone imbalances [11–13]. Finally, MetS was also considered a low-grade systematic inflammatory state that may enable the body to release more subclinical inflammatory factors, such as IL-6, CRP and TNF-α [14–17]. Interestingly, although a systematic study has not been carried out so far, individual studies have shown that the abovementioned major pathogenic factors involved in MetS may play a role in serum PSA levels [18–20].

As the underlying mechanism connecting MetS to PSA levels is complicated and undefined, there is a compelling need for further understanding these topics. Therefore, the aim of the present study was to clarify the underlying mechanism connecting MetS to serum PSA levels by using multivariate regression models.

## Methods

From October 2014 to August 2015, by routine physical examination programs, 506 men over 45 years old were recruited consecutively to the study. By completing a standardized, structured questionnaire, subjects who were diagnosed with urethritis and prostatitis, urethral stricture, and neurogenic bladder, and who had a history of urological surgery or trauma and malignant diseases of the urinary system were excluded from this study. Additionally, subjects were also excluded if they had received anticholinergics, 5α-reductase inhibitors or hormone replacement therapy. These study protocols were approved by the the ethics committee of Beijing shijitan hospital affiliated to capital medical university and China railway corporation, and all subjects received informed consent and agreed in writing prior to registration.

All subjects completed the International Prostate Symptom Sore (I-PSS) questionnaire after Chinese translation, and the medical history of subjects was collected using a standardized, structured questionnaire. Anthropometric measurements, including blood pressure (mmHg), body weight (kg), height (cm) and waist circumference (cm), were measured by trained nurses using a standardized protocol. Body mass index (BMI) was calculated by using the formula (Square of weight/height). The PV (cm3) was measured by suprapubic ultrasonography (3.5 MHz, Hitachi EUB-400, Tokyo, Japan) using the formula for the volume of an ellipse (height×width×length×π/6).

Before undergoing direct recording electronic, a 10-mL, 12-h fasting blood specimen was drawn for biochemical analyses after the participants had been relaxed sitting for 15 min. The biochemical index analysis included PSA, fasting blood glucose (FBG), triglycerides, low-density lipoprotein cholesterol (LDL-C), high-density lipoprotein cholesterol (HDL-C) and total cholesterol. Insulin, 5α-dihydrotestosterone (DHT), sex hormone-binding globulin (SHBG), estradiol, testosterone, leptin, resistin, adiponectin, Il-6, CRP and TNF-α were measured using enzyme-linked immunosorbent assay methods. The IR index was derived using the HOMA arithmetic: FBG (mg/dL) × Insulin (μU/mL)/405 [21].

We defined MetS using the criteria established by the 2009 joint statement from the International Diabetes Federation Task Force on Epidemiology and Prevention; National Heart, Lung and Blood Institute; American Heart Association; World Heart Federation; International Atherosclerosis Society; and International Association for the Study of Obesity [4]. According to that report, MetS was diagnosed based on the simultaneous occurrence of at least three of the following five risk factors: waist circumference ≥ 90 cm; triglyceride levels≥150 mg/dL or drug treatment for elevated triglyceride levels; HDL cholesterol≤40 mg/dL or drug treatment for low HDL cholesterol; elevated blood pressure (systolic blood pressure ≥ 130 mmHg, diastolic blood pressure ≥ 85 mmHg or antihypertensive drug treatment with a history of hypertension); and FBG levels≥100 mg/dL or drug treatment for elevated FBG levels.

Continuous variables are shown as the mean ± SD, and categorical variables are shown as numbers and percentages. We divided subjects into two groups based on the presence of MetS. The clinical characteristics were compared using an independent t test for continuous variables and a chi-square test for categorical variables. Multivariate linear regression models were used to evaluate the effect of MetS-related factors on serum PSA levels. Furthermore, we divided subjects into four groups based on the quartile serum levels of insulin and SHBG to investigate whether there was a linear difference in the mean PSA level among the groups by using ANOVA. Data were analyzed using SPSS software version 13.0 for Windows (SPSS Inc., Chicago, IL, USA), and two-tailed *P* values < 0.05 were considered significant.

## Results

The baseline characteristics of the subjects are presented in Table 1. The mean age was 69.3 ± 8.3 years. The overall prevalence of MetS in the entire cohort was 37.5% (190/506). Age, BMI, waist circumference, blood pressure (which included the systolic pressure and diastolic pressure), FBG, triglycerides, PV, IPSS score, HOMA index and leptin levels were significantly higher in subjects with MetS than in subjects without MetS (all *P* < 0.01). HDL-C, TNF-α and testosterone levels were significantly lower in subjects with MetS than in subjects without MetS (all *P* < 0.05).

**Table 1:** Baseline characteristics

Characteristics	Overall(506)	Mets(190)	non-Mets(316)	*P*-value
Age	69.27 ± 8.31	70.63 ± 7.93	68.46 ± 8.44	**0.004†**
BMI, kg/m^2^	24.70 ± 3.10	25.26 ± 2.93	24.36 ± 3.16	**0.002†**
Waist circumference, cm	86.91 ± 8.69	89.00 ± 8.44	85.66 ± 8.61	**<0.001†**
SBP, mmHg	137.61 ± 15.52	146.74 ± 13.05	132.13 ± 14.27	**<0.001†**
DBP, mmHg	79.22 ± 10.56	81.77 ± 10.65	77.69 ± 10.22	**<0.001†**
Elevated blood pressure, *n* (%)	72.1	95.8	50.1	**<0.001‡**
FBG, mg/dL	102.14 ± 25.45	113.04 ± 31.37	95.59 ± 18.25	**<0.001†**
Triglycerides,mg/dL	160.38 ± 138.81	225.63 ± 191.67	121.15 ± 68.81	**<0.001†**
HDL-C, mg/dL	47.29 ± 11.45	40.20 ± 8.65	51.56 ± 10.80	**<0.001†**
LDL-C, mg/dL	111.86 ± 30.59	109.86 ± 29.27	113.07 ± 31.34	0.253†
PV, cm^3^	24.57 ± 7.15	26.31 ± 7.83	23.53 ± 6.50	**<0.001†**
Q_max_, mL/s	15.41 ± 6.09	11.96 ± 4.31	17.50 ± 6.07	**<0.001†**
PSA, ng/mL	1.35 ± 1.12	1.36 ± 1.10	1.35 ± 1.14	0.940†
IPSS	13.04 ± 7.62	14.26 ± 7.34	12.30 ± 7.71	**0.005†**
Insulin, pmol/L	51.68 ± 31.08	52..93 ± 29.74	50.93 ± 31.88	0.484†
HOMA IR	2.15 ± 1.35	2.42 ± 1.52	1.98 ± 1.21	**<0.001†**
Leptin, ng/mL	3.51 ± 2.83	4.21 ± 2.79	3.09 ± 2.77	**<0.001†**
Resistin, ng/mL	29.70 ± 18.29	29.05 ± 16.39	30.09 ± 19.35	0.518†
Adiponectin, μg/mL	2.99 ± 1.70	2.99 ± 1.65	2.99 ± 1.72	0.991†
CRP, mg/L	2.20 ± 4.31	2.24 ± 3.58	2.18 ± 4.70	0.871†
IL-6, pg/mL	1.87 ± 5.47	1.97 ± 7.44	1.81 ± 3.84	0.755†
TNF-α, pg/mL	43.52 ± 9.63	42.20 ± 9.41	44.31 ± 9.69	**0.017†**
DHT, pg/mL	380.87 ± 230.30	370.63 ± 208.30	387.05 ± 242.73	0.438†
SHBG, nmol/L	70.71 ± 35.21	67.39 ± 33.34	72.72 ± 36.20	0.100†
Estradiol, pg/mL	38.70 ± 17.10	40.29 ± 19.50	37.74 ± 15.43	0.117†
Testosterone, ng/mL	4.61 ± 1.75	4.31 ± 1.36	4.79 ± 1.92	**0.001†**
E2/T	9.67 ± 8.66	10.23 ± 5.89	9.33 ± 9.96	0.261†
Number of MetS components, *n*				
0	–		48 (9.5%)	–
1	–		126 (24.9%)	–
2	–		142 (28.1%)	–
3	–	116 (22.9%)		–
4	–	63 (12.5%)		–
5	–	11 (2.2%)		–

*MetS* Metabolic syndrome, *BMI* Body mass index, *SBP* Systolic blood pressure, *DBP* Diastolic blood pressure, *FBG* Fasting blood glucose, *HDL* High-density lipoprotein, *LDL* Low-density lipoprotein, *PV* Prostate volume, *CRP* C-reactive protein, *IL-6* Interleukin 6, *TNF-α* Tumor Necrosis Factor alpha, *DHT* 5α-Dihydrotestosterone, *SHBG* Sex hormone binding globulin; *P* were calculated by independent t-test† and chi-squared test‡; Bold indicates statistically significant values

In Table 2, results shows that among the MetS components only HDL-C was positively and significantly correlated with serum PSA levels after adjusting for age, BMI and PV (*P* = 0.046).

**Table 2:** Relationship between MetS and PSA

Characteristics	B	*t*	95% CI	†*P*-value
Waist circumference, cm	− 0.043	− 0,444	− 0.232, 0.147	0.657
Triglycerides, mg/dL	− 0.026	− 0.567	− 0.114, 0.063	0.571
HDL-C, mg/dL	0.089	1.998	0.001, 0.177	**0.046**
SBP, mmHg	0.080	1.673	−0.014, 0.173	0.095
DBP, mmHg	0.001	0.002	−0.091, 0.091	0.998
FBG, mg/dL	0.015	0.339	−0.073, 0.103	0.735
MetS	0.052	0.547	−0.134, 0.238	0.584

*CI* Confidence interval, *SBP* Systolic blood pressure, *DBP* Diastolic blood pressure, *FBG* Fasting blood glucose, *HDL* High-density lipoprotein; †*P*-value were calculated according to Multivariate linear regression analyses; Bold indicates statistically significant values

As shown in Table 3, after adjustment with age, BMI, PV and HDL-C in separate multivariate linear regression models, the data showed that serum insulin levels, SHBG and serum testosterone levels were significantly and linearly correlated with PSA levels (all *P* < 0.05). Serum testosterone levels were positively associated with serum PSA levels, while serum insulin levels and SHBG were negatively associated with serum PSA levels. Interestingly, when serum insulin levels, SHBG and serum testosterone levels were simultaneously adjusted in addition to adjusting age, BMI, PV and HDL-C, serum insulin and SHBG levels still showed significant and negative relationships with serum PSA levels (both *P* < 0.05).

**Table 3:** Analysis of MetS-related affect PSA

Characteristics	B	95% CI	†*P*-value	B	95% CI	‡*P*-value
Insulin	−0.241	− 0.492, 0.009	**0.049**	− 0.172	−0.272, -0.073	**<0.001**
HOMA index	0.076	−0.169, 0.321	0.542			
DHT	−0.089	−0.184, 0.006	0.068			
SHBG	−0.182	−0.305, -0.058	**0.004**	−0.163	− 0.284, -0.042	**<0.001**
Estradiol	0.062	−0.028, 0.153	0.177			
Testosterone	0.128	0.006, 0.251	**0.039**	0.083	−0.032, 0.198	0.158
Leptin	−0.033	0.159, 0.093	0.607			
Resistin	0.061	−0.030, 0.153	0.186			
Adiponectin	0.041	−0.058, 0.140	0.412			
CRP	0.032	−0.064, 0.129	0.510			
IL-6	−0.072	−0.175, 0.031	0.169			
TNF-α	−0.025	−0.122, 0.072	0.617			

Abbreviations as in Table 1 and Table 2

†*P*-values were separately calculated three different statistics model according to multivariate linear regression analyses after adjustment of age, BMI, prostate volume and HDL-C;The model 1 include Insulin and HOMA index;The model 2 include DHT, SHBG, Estradiol, Testosterone; The model 3 include Leptin, Resistin, Adiponectin, CRP, IL-6, TNF-α;

‡*P*-value were calculated statistics model include Insulin, SHBG, Testosterone according to multivariate linear regression analyses after adjustment of age, BMI, prostate volume and HDL-C;

Bold indicates statistically significant

**Fig. 1:**
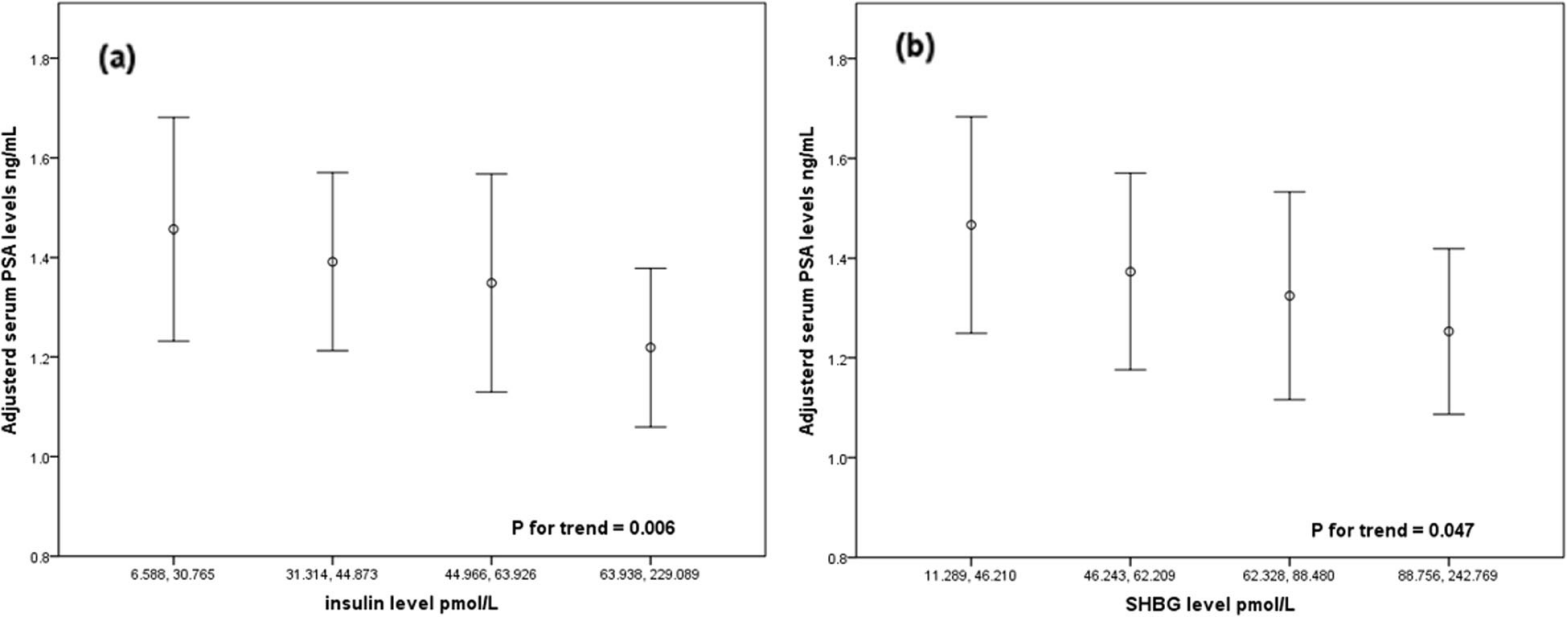
Adjustment with age, BMI, PV and HDL-C, the mean PSA level decreased as (**a**) serum insulin and (**b**) SHBG levels increased

Finally, we divided subjects into four groups based on the quartile serum levels of insulin and SHBG to investigate whether there were differences in the trend of serum PSA levels among the groups (Fig. 1). After adjusting for age, BMI, PV and HDL-C, the mean PSA level significantly and gradually decreased as serum insulin and SHBG levels increased (both P for this trend < 0.05).

## Discussion

The results of the study indicated that insulin resistance and SHBG levels play a important role in the lower serum PSA levels in MetS patients. Previously, researchers have noted that the presence of MetS is associated with the risk of PCa development, but there was controversy over the correlation between MetS and the risk of PCa [22, 23]. The heterogeneity of previous research conclusions among different studies may be caused by the differences in sample sizes, baseline characteristics, MetS diagnostic criteria and follow-up time. However, considering the influence of MetS on the serum PSA level, this may be one of the reasons for the deviations in the correlation between MetS and the risk of PCa.

A study noted that although screening serum PSA levels can improve the detection rate of PCa, it has a relatively small impact on PCa-specific mortality, and researchers have suggested that when screening serum PSA levels, it is necessary to fully consider the problems of missed diagnosis and overdiagnosis [3]. The causes of these problems may involve the excessive interference of serum PSA levels.

In a study by Choi et al. [7], the serum PSA levels in the MetS group were lower than those in the control group (1.15 ± 0.91 vs. 1.26 ± 0.76 ng/ml, *P* = 0.006), and after adjusting for PV and plasma volume, the results were also significantly correlated. Research has suggested that MetS is an independent risk factor for decreased serum PSA levels (serum PSA levels fell by 4.1%, *P* = 0.046). A recent study from our research team demonstrated that serum PSA levels in the MetS group were lower than those in the non-MetS group (1.11 ± 0.79 vs. 1.21 ± 0.76 ng/ml, *P* = 0.026) and that MetS was an independent risk factor for a decrease in serum PSA levels, which can reduce the serum PSA level by 11.3%. We also reported that as the number of abnormal components in MetS increases, the serum PSA level presents a trend of progressive decline. This study was based on a sample of 45,540 healthy men aged 55 to 59 [9]. However, the serum PSA level was not associated with MetS in our study, which may have been due to the small sample size. However, based on our previous studies with large samples, it MetS is believed to be correlated with PSA declines.

A considerable number of studies have confirmed that MetS aggravates the degree of IR, and IR is not only the driving factor of MetS but is also one of the main pathogenic factors [10]. In a study of Parekh et al. on the relationship between physiological indicators and serum PSA concentration, their results showed that the serum PSA concentration was significantly and negatively correlated with IR (*p* = 0.04) after adjusting for age, BMI, and race [18]; Choi et al. [6] reached a similar conclusion in their study. However, previous studies failed to consider the large PV of MetS patients. Our results indicated a significant and negative association between insulin concentrations and PSA (*P* = 0.049) after adjustment with age, BMI, PV and HDL-C. Furthermore, the mean serum PSA levels showed a significantly declining trend with increasing insulin levels, as presented in Fig. 1a (P for trend =0.006). We believe that changes in the insulin level caused by IR are one of the pathogenic factors that play a crucial role in the influence of MetS on serum PSA levels.

Sofikerim et al. [24] analyzed 210 subject whose serum PSA level was less than 2.5 ng/ml and pointed out that there was no significant correlation between the serum PSA level with the serum testosterone and serum free testosterone concentration. A similar study demonstrated that there was no linear correlation between the serum testosterone concentration and the serum PSA level, and testosterone replacement therapy did not cause changes in the serum PSA level [25]. It is worth noting that Rastrelli et al. indicated a strong positive correlation between testosterone with PSA levels in subject with low testosterone; additionally, a similar idea was proposed in a previous study, in which researchers suggested that a natural decrease in serum androgen levels might alter PSA levels [26, 27]. Conversely, several studies have found a correlation between SHBG and the risk of MetS; as a carrier that combines and transports sexual steroids, changes in the SHBG level may better reflect changes in sexual hormone levels [11–13]. Considering the interaction between sex hormones, estradiol and DHT were also taken into account when we analyzed these data. Our results showed that the testosterone levels were positively associated with PSA levels after adjustment with age, BMI, PV and HDL-C, while SHBG was negatively correlated with serum PSA levels in the sex hormone factor group (*P* = 0.039, *P* = 0.004, respectively). The mean PSA levels tended to decrease as the concentration of SHBG increased (P for trend =0.047), as shown in Fig. 1b. We found that SHBG levels could be a better indicator than testosterone and estrogen, which should be considered further when examining the MetS-PSA relationship.

The pathology caused by MetS leads to chronic inflammation and changes in the serum levels of inflammatory factors and adipokines, including leptin, adiponectin, CRP, IL-6, TNF-α, etc. [14–17]. McDonald et al. found a correlation between serum CRP levels and elevated PSA levels (OR = 1.19; 95% CI, 1.06–1.33), suggesting that systemic inflammatory markers are associated with PSA elevation in the absence of prostate disease [19]. However, there was no significant association between serum leptin and adiponectin with PSA levels in previous studies [20, 28]. We found no association between subclinical inflammatory mediators and serum PSA levels, but the current results do not completely negate the influence of systemic inflammation on serum PSA levels, which may be caused by the interaction of inflammatory markers, such as leptin and adiponectin. Thus, we still need to conduct more detailed research and analysis to further clarify the correlation between serum PSA and inflammatory mediators.

Finally, indicators that had a significant correlation with the serum PSA level in previous studies were included in the multiple linear regression model after adjustment with for age, BMI, PV, HDL-C. There was a significant, negative correlated between insulin and SHBG with serum PSA levels (all *P* < 0.001). Previous research has indicated that SHBG are negatively correlated with glycosylated hemoglobin in people without diabetes, which indicates that there may be a relationship between SHBG and glucose homeostasis changes before diabetes [29]. Similar researches have found that there is a negative correlation between SHBG with insulin levels, insulin resistance, and diabetes risk, and researchers believe that low serum SHBG is an independent risk factor for type 2 diabetes [30]. Although the relationship between SHBG and insulin resistance needs to be further explored, it can be concluded that both insulin resistance and SHBG levels play a crucial role in the changes in serum PSA levels in MetS patients.

There are still some limitations in the present study because MetS leads to considerable, complicated pathophysiologic changes. However, according to existing research results, we narrowed the scope of this study. Due to the interaction between pathogenic factors, the current results may ignore the impact of a single pathogenic factor, so we still need to further explore the influence of single pathogenic factors on serum PSA levels. In addition, since prostate biopsy was not included in our health examination plan, we cannot explicitly exclude the existence of prostate cancer, but because the average PSA levels of our target population were low, even a small number of patients with early PCa would not affect the results of the current study.

## Conclusions

The decrease in serum PSA levels in MetS patients is mainly related to insulin resistance and serum SHBG levels. Urologists need to pay attention to this effect to comprehensively evaluate patient serum PSA levels, and subsequent studies need to further refine the serum PSA correction formula for MetS patients based on research results and to explore the physiological mechanism of this process.

## Data Availability

The datasets used and/or analysed during the current study are available from the corresponding author on reasonable request.

## Abbreviations

BMI: Body mass index
CRP: C-reactive protein
DHT: 5α-Dihydrotestosterone
FBG: Fasting blood glucose
HDL-C: High-density lipoprotein cholesterol
LDL: Low-density lipoprotein cholesterol
MetS: Metabolic syndrome
Pca: Prostate cancer
PSA: Prostate-specific antigen
PV: Prostate volume
SHBG: Sex hormone binding globulin
